# Supplementary material for: Subjective and psychophysiological response to pictures of ancestral and modern threats: Not all evolutionary threats are alike
Source: PLoS One. 2026 Mar 18;21(3):e0343680. doi: 10.1371/journal.pone.0343680 (PMC12998808; doi:10.1371/journal.pone.0343680)
Supplement: Attachment — Submitted filename: Review_PLoS_Physiology.docx [file pone.0343680.s013.docx]

**Reviewer #1**

*The paper describes a study on the physiological reactions to ancestrally- vs culturally-feared stimuli. The sample is larger than the average of this research line; all procedures and data are exhaustively presented and shared; the introduction and discussion are in general coherent with the results. Broadly speaking, the article is of good quality, and aims at disambiguating a research question which led to controversial findings.*

Thank you for your kind words.

*Despite the generally-good quality of the paper, I have some concerns about the Introduction and the Discussion sections:*

*• the introduction is quite verbose due to an exhaustive specification of the nature/nurture evidences concerning each of the stimulus categories (i.e., snakes, guns...). While I agree with the necessity to elucidate such information, I find it too long and specific to be written in an introduction: the decisional process which led to choose these stimuli is rightfully pertinent to the introduction, but such level of detail is not. Thus, the solution I propose is to transfer in a supplementary table or chapter all the detailed information about all the studies supporting the nature/nurture debate on each stimulus category, leaving in the introduction few more general sentences summarizing the choice of some ancestrally-feared and of some culturally-feared stimuli.*

**Accepted.** We substantially restructured and shortened the introduction in accordance with your recommendations as well as recommendations of Reviewer #2.

*On the other hand, the space saved in the introduction should be partially filled with some little discussion of the scientific debate on subliminally-induced skin conductance reactions in response to the same kinds of stimuli (ancestral or cultural): this is only slightly mentioned so far, while I would find it very relevant to introduce (and, later, discuss) your results. Indeed, the authors did a great job recruiting 119 participants and showing them this variety of pictures, but these results alone cannot give a totally exhaustive answer about the nature/nurture picture processing debate.*

**Accepted.** We have substantially revised both the Introduction and the Discussion in light of your comments, specifically added subliminally-induced skin conductance research and also newly introduced Limitations to address your concerns (see also below).

*This leads me to the concerns about the Discussion; coherently with the results, it is discussed that there is not much difference between ancestral and cultural picture processing, with the only exception of height pictures. However, why heights and not – e.g. – snakes? They are both ancestrally-feared stimuli that should be expected to elicit a comparable reactivity: please detail this better.*

**Accepted.** We significantly extended this part of discussion, laying more evidence that might explain the observed differences (Lines 505-550).

*More importantly, these results concern the skin reactions to pictures lasting many seconds: during this time, lot of things could happen. For example, an initial reaction could be stronger in the first (milli)seconds for ancestral stimuli, while a latter thinking on culturally-feared stimuli could elicit a delayed reaction: this would make a difference at the beginning still yielding no significant differences on a larger window. It is not clear to me whether this possibility can be unambiguously excluded based on the statistical analyses, which seems to not deeply analyze temporal components. If you did not address the temporal facets of skin reaction, this is still ok but should be mentioned as a potential limitation while discussing the results.*

**Partially accepted.** While this is a very intriguing idea, measurements of skin conductance / skin resistance do not offer the temporal precision required for such analyses. Although our data were sampled at 500 Hz, which makes the inspection of very short time windows (e.g., 10 ms) technically possible, current knowledge of the regulatory mechanisms underlying emotional sweating indicates that such analyses would be, at best, dominated by artefacts and, at worst, scientifically meaningless. The key issue is the delay of the skin resistance response, which typically occurs 1–3 seconds after stimulus onset (Boucsein et al., 2012). This long and variable delay restricts meaningful analyses to longer or medium-long intervals. As recommended, we have also added the method’s limited temporal accuracy to the Limitations section.

Boucsein, W. et al. Publication recommendations for electrodermal measurements. Psychophysiology 49, 1017–1034 (2012). https://doi.org/10.1111/j.1469-8986.2012.01384.x

*Indeed, to really exclude the existence of a difference between the processing of ancestrally-feared and culturally-feared stimuli, you should also have administered the same pictures subliminally. This was not probably feasible in the timing of the study, and it is ok to present only results about clearly-visible stimuli: but should nevertheless described as a potential limitation in the answer to the research questions of interest, and suggested as a future development of the study. This issue also concerns the relatively-simple analysis performed on skin conductance data, which is ok but could be deepened with more exhaustive approaches: this could be also mentioned as a potential limitation and as an invitation for other researchers to perform other analyses on the shared data.*

**Accepted.** We have added a section titled “Limitations and future directions”, which addresses these concerns and suggestions. As is customary, this section appears at the end of the Discussion, immediately before the Conclusions.

*Other minor issues concern:*

*• the full availability of data. I really appreciated the data shared by the authors in form of supplementary tables, however I would like them to upload everything (including both the raw and pre-processed electrodermal data, and the R/Matlab scripts used to analyze them) on some open repository (e.g., Open Science Framework) for a better replication of the study and re-analysis of its results*

**Partially accepted.** We now provide the raw data for all participants together with a basic description of the algorithm used to process them and extract the variables we analyse (number of responses, response amplitude and response latency). However, we cannot share the script itself because we do not own it. Although it was developed in coordination with our team, the original developers retain the rights to their product, and we are therefore not permitted to make the algorithm public. The raw data are now available via https://doi.org/10.5281/zenodo.17722322.

*• some typos of minor importance (e.g., uppercase after semicolon in line 295) which nevertheless should be carefully checked and corrected. Also, I see the figures with quite a low quality, which is still intelligible but I hope that the final version will be of better quality.*

**Accepted.** We carefully went through the text word-by-word and checked the language issues raised by both you and reviewer #2.

*After addressing all these points (including the full sharing of data), I will see no reasons to not endorse this interesting paper.*

Thank you for the opportunity to revise the manuscript.

**Reviewer #2**

*In this work, Štolhoferová, et al. aim to elucidate physiological responses through skin conductance on stimuli representing ontogenetic and phylogenetic threats. Additionally, they investigate the bilateral relationship between physiological responses and subjective measurements. I appreciate this interesting avenue of research and the efforts of the team involved to acquire over a hundred subjects in a few months. However, I have serious doubts regarding the experimental setup, the rigour, and suitability of statistical analysis. I think the manuscript needs to be thoroughly revised to highlight the contextual importance of stimuli, rather than finding a dichotomy between ancestral and modern threats. Specifically, the fact the even snakes – a canonical “fearful” stimulus – does not elicit a strong response, suggests contextual processes are ongoing. I think the manuscript would have to argue along the lines of such selective embodiment.*

Thank you for the opportunity to revise the manuscript. During the revision process, we aimed to focus on providing deeper background, more thorough analysis and better contextualization of our findings. Hopefully, these changes will improve the quality of the manuscript.

*MAJOR POINTS*

*1. The major aim of the study seems to be the differentiation of particular categories of threats (ancient vs modern) based on skin conductance responses. Most stimuli included are questionably attributable to either class. For instance, even though “guns” are a modern concept (and thus a learned association with danger), the underlying circuits that drive the signal-of-interest may still be rooted in evolutionary mechanisms. As such, babies seem to be quite curious around snakes, suggesting there is a similar learned association. Similar arguments can be made for the other stimuli, rendering a hard dichtomy between ancient and modern threats rather difficult. All stimuli, therefore, probably activate the same domain-general salience network in the brain. It will care about how close the threat is, how probable damage to the body is, and how controllable the threat is.*

**Explained.** We agree that there is no strict dichotomy between ancient and modern threats in sense that both types of stimuli engage largely overlapping neural networks and structures involved in the perception, processing and regulation of threat-related responses, regardless of their evolutionary origins. The resulting emotional, physiological and behavioural responses are shared to an even greater extent. We did not hypothesise that ancient and modern threats activate entirely distinct neural circuits leading to fundamentally different physiological pathways or responses. As you correctly note, current evidence indicates that the neural circuits underlying threat processing are broadly shared across threat categories. Nevertheless, their activation and deactivation patterns, as well as their connectivity, may differ systematically. We have now addressed this point more thoroughly in the revised Introduction (Lines 69-105). In light of those findings, we consider it worthwhile to examine potential differences in physiological responses to different types of threats. However, we do not expect entirely distinct reaction profiles for each threat type, but rather mean differences in one or more of three basic parameters of the response: its probability, amplitude (saliency) or latency (newly added analyses). In the revised manuscript, we have aimed to clarify the study’s aims, its position within the current state of knowledge and the ways in which it meaningfully advances it.

*2. Given that personal, cultural, and social factors play such a large role in shaping the fear response, I do not think a between-subjects design is the most appropriate. I do understand that between-subjects design results in less carry-over, but I think controlling for individual differences outweighs the possibility that responses to one stimulus might affect the next. This might also underlie the result that only heights seem to really induce a physiological response.*

**Explained.** This is indeed a complex issue, and we appreciate your point, as there can be considerable inter-individual differences in reactivity among participants. The key to controlling for such variability lies in the fact that all participants were exposed to the same control stimuli, and that we employed mixed-effect models for data analysis. These models incorporate participant ID as a random factor and assume that responses obtained from a single participant are inherently more similar to each other than responses obtained from different participants. Consequently, if one participant happens to be highly reactive while several others are less so, the final mean across participants is less influenced by this “outlier” individual than it would be under a simple arithmetic mean. Moreover, for determining statistical significance, mixed-effect models operate on a matched-matrix principle, effectively comparing responses to control and threat stimuli within each individual and computing the final statistics from these repeated, independent comparisons.

Nonetheless, we acknowledge that a within-subject design would provide a more direct comparison between mean responses to threat stimuli. Our choice of a between-subject design was driven primarily by stimulus variability and presentation length. Based on our experience, extended presentation sessions tend to yield unreliable data because participants find it difficult to maintain concentration and minimise movement, which leads to the automatic exclusion of potential responses. While stimuli such as snakes and guns are relatively straightforward—since the “concept” of a snake or gun can be adequately represented by just seven images (28/4, or the total number of threat stimuli divided by the number of threat categories)—this is not true for stimuli such as heights or pandemics. These are more complex and abstract concepts, particularly when restricted to image-based presentation. We were therefore not confident that a limited number of images (seven to ten) would sufficiently capture the essence of “the threat of heights” or “the threat of a pandemic”. Increasing the number of images per category, however, would have extended the presentation time over acceptable length. In retrospect, using around ten images per threat category would have probably been sufficient, and a within-subject design would have allowed a more direct comparison between threats. At the moment, though, we can only note this consideration among the study’s limitations and future directions.

*3. The statistical analyses mainly depend on GLMMs and AICs. In light of the obscure nature of the stimuli, I would have like to see clustering approaches to identify how stimuli relate to one another. Now the reader is presented with what seems to be an “odd” result (only height seems to drive a response). Specifically, the fact that snakes elicited such a weak response should have prompted more in-depth analyses. Rather than digging in further into how this may have arisen, the reader is presented with a broad “what even is fear” discussion.*

**Partially accepted.** We performed the analysis using k-means clustering, as you suggested below. We also revised the discussion to better clarify our reasoning behind the results. However, the various explanations for the exceptionally frequent and salient responses to heights are largely theory-based, and in some cases somewhat speculative, which is why we have kept them brief. We would be happy to include a more detailed, empirically supported explanation if you could kindly point us in the right direction.

*4. The introduction/discussion in general are fragmented: each stimulus type received a paragraph as to why the obtained results could have made sense. I am missing a discussion on the overlap of stimuli, shared circuitry, and how personal/contextual cues might shape the responses.*

**Accepted.** We have substantially revised both the Introduction and the Discussion in light of your comments, and we hope that the revised version addresses your concerns more effectively.

*MINOR POINTS*

*1. I would have liked a more in-depth discussion on how the skin resistance signal arises. This is now briefly touched upon in line 47-49, but could be expanded to aid the novices to skin condutance. Moreover, the authors may want to strengthen the justification for EDA over other modalities that are linked to arousal (e.g., pupil dynamics, heart period responses, etc), or even combine them (in future studies).*

**Accepted.** We included this information in the Introduction, lines 119-137.

*2. The visualizations of this study are rather sparse. I am only getting to see summary/descriptive statistics, but no actual skin conductance traces. This removes me far from the actual data.*

**Accepted.** We added an example of raw data as Figure 2.

*3. In Figure 2b, the different letters “indexes unsignificant differences between categories”. The table in Table S5b shows that the leaves and airborne disease categories do not significantly differ. Why, then, do they have different indices? Am I looking in the wrong table? Or is “leaves” always annotated with “a” as it’s the control condition?*

**Accepted.** This was a mistake from our part, thank you for pointing it out.

*4. Figure 3 requires uncertainty measures around the fit. It is unlikely that the distribution of rating values is homogeneous across ratings (due to central tendency or floor effects) (See Figure S1). The extreme linearity is hard to interpret without a sense of variability.*

**Accepted.** We adjusted the figure and added 90 % CIs around the lines (conventional 95 % CIs were too wide and the figure was difficult to read).

*5. Line 143 reads “Nonetheless, it is not straightforward as it might seem to estimate how long airborne diseases have …”. I fail to see why this would be straightforward.*

**Accepted.** We adjusted the wording.

6. Line 231: it is easier to just refer to “participants”

**Accepted.**

*7. Line 241: the stimuli alternated? Does this mean there was an absence of randomization? This would induce quite some predictive processes going on, as the stimulus sequence is predictable.*

**Explained.** The presentation order was semi-randomized, with each threat stimulus always followed by a control stimulus and *vice versa*. Although responses due to predictive processing are theoretically possible, we are confident that we minimised them during both data collection and curation. First, after each stimulus (control or threat), a black screen was presented for at least 5 seconds. During this interval, the researcher monitored the SR response in real time and only allowed the next stimulus to appear once the SR curve had flattened and approximately returned to baseline. Furthermore, during data curation, we excluded all SR responses that peaked within the first second after stimulus onset. This approach is recommended in the literature (Boucsein et al., 2012), as SR changes are detectable only after approximately 1000 ms; any earlier responses thus likely reflect anticipation rather than reactions to the presented stimulus. We provided more detailed explanation of this in the methods.

Boucsein, W. et al. Publication recommendations for electrodermal measurements. Psychophysiology 49, 1017–1034 (2012). https://doi.org/10.1111/j.1469-8986.2012.01384.x

*8. Line 260: not a critique, but 18-74 is a broad age range!*

Yes, in recent years we have aimed to include older participants in our experiments so that the sample better approximates the general population.

*9. Line 261: did the power analysis also include the between-subjects structure of the experiment?*

**Explained.** Yes, it did.

*10. Line 291: given the potential bottom-up versus top-down influences to different stimuli, it would actually be interesting to say something about the response latencies. Also with respect to the non-random nature of the experiment, one may expect responses before the stimulus onset.*

**Accepted.** We newly added analysis of response latencies, however, measurements of skin conductance / skin resistance do not offer very good temporal precision. Although our data were sampled at 500 Hz, which makes the inspection of very short time windows (e.g., 10 ms) technically possible, current knowledge of the regulatory mechanisms underlying emotional sweating indicates that such analyses would be, at best, dominated by artefacts and, at worst, scientifically meaningless. The key issue is the delay of the skin resistance response, which typically occurs 1–3 seconds after stimulus onset (Boucsein et al., 2012). This long and variable delay restricts meaningful analyses to longer or medium-long intervals. Nonetheless, we performed at least basic analysis of response latency as suggested. Regarding the responses anchored in anticipation, please view the response to question 7.

Boucsein, W. et al. Publication recommendations for electrodermal measurements. Psychophysiology 49, 1017–1034 (2012). https://doi.org/10.1111/j.1469-8986.2012.01384.x

*11. Given the subtle effects that seem to be at play, I would like to see more uncertainty measures (if AIC) produces something like that (e.g., confidence intervals, Cohen’s D).*

**Accepted.** We have added effect size measures (R²) and ensured that confidence intervals are included wherever possible. Regarding the AIC, it does not provide a measure of uncertainty because it is highly context-dependent and is intended only for comparing models that differ in additive effects. Therefore, the absolute value of the AIC is less important than whether one model’s AIC is higher or lower than another’s.

*12. I think figure 4 requires more discussion as to what it implicates. Which ratings drive the effect? A strong reduction of rating = 1, but where do these other ratings go? Uniformly to the other score or generally towards higher ratings?*

**Explained.** No, the difference is not uniformly distributed—any six of the levels are treated as independent, and only the seventh is defined as 100% minus the sum of the others. Unfortunately, there is no simple way to describe each change, although we added a description of the overall trend in the figure caption. Nonetheless, we would like to advise caution when interpreting this figure and the model on which it is based, as the underlying computations are extremely demanding and there is considerable uncertainty in the estimated model parameters and while the overall message is sound, extensive details might be trickier.

*13. In section 3.5, did you control for the number of tests (i.e., number of questionnaires) or were they all considered to be independent samples?*

**Explained.** These were all considered independent samples since data of only those participants who viewed the specific type of threat are analysed in each test.

*14. Line 537: can you show this interstimulus separation?*

Unfortunately, we were not able to find any mention of interstimulus separation on this line or anywhere near it. Perhaps there was a typo?

*15. Grammatical/sentence structure is sometimes a bit complex:*

*a. Line 99: Restructure to “… these stimuli [40,41]. For example, …”*

*b. Lines 104-107 are hard to parse*

*c. Lines 127-130 are also hard to parse*

*d. Line 473: “bring” should be “brought”*

*e. Line 512: “though” should be “through”*

*f. I may have missed other typos*

**Accepted.** We carefully went through the text word-by-word and checked the language issues raised by both you and Reviewer #1.

16. I applaud the authors for sharing all the data. However, it would be great if the accompanying code could be shared to. This should include documentation on how to use the code, so other researchers (including me) can learn more from this data.

**Partially accepted.** Similar inquiry was also made by Reviewer #1 therefore we now provide the raw data for all participants together with a basic description of the algorithm used to process them and extract the variables we analyse (number of responses, response amplitude and response latency). However, we cannot share the script itself because we do not own it. Although it was developed in coordination with our team, the original developers retain the rights to their product, and we are therefore not permitted to make the algorithm public. The raw data are now available via https://doi.org/10.5281/zenodo.17722322.

*17. After manuscript revision, the authors may reconsider the title of the manuscript. It now focuses on the fact that among evolutionary responses, only heights evokes a strong response compared to snakes. I think the title should go more in the direction of unified/selective embodied representations of this fear-spectrum.*

**Declined.** We are fond of the current and in our view, it actually reflects the heterogeneity of “evolutionary” versus “modern” threats, therefore we opted to keep it as is.

*TECHNICAL SUGGESTIONS*

*1. For clustering approaches, one could think of k-Means clustering or support vector machines to try and elucidate particular clustering of stimulus types. This may either show an expected evolutionary progression, or it may be inseparable, which would be evidence for a domain-general mechanism.*

**Accepted.** We have added this analysis to the Supplementary files because, in our view, the results do not strongly support either of the hypotheses you mention. This may simply reflect the fact that only a limited number of variables can be extracted from skin resistance measurements, leaving the analysis with relatively little to work with. This is also one of the reasons why our aims were more modest and why we prefer to analyse the three parameters separately.

*2. As salience is such an important factor in studies like this, it may be valuable investigating the stimulus energy of the presented images across stimulus types. This may say something about how much each stimulus captures attention.*

Could you please rephrase this suggestion? Are you referring to valence ratings, or to low-level visual features of the stimuli (colour, luminance, orientation, etc.)? And regarding attention capture, are you suggesting that some stimuli might not have received sufficient attention even if participants looked at them and were instructed to free-view them?

*CONCLUDING REMARKS*

*Again, I would like to commend the authors for their hard work and I apologize if my tone was harsh. With a major revision of the framework, updated visualizations, and additional statistical tests, I feel this work can provide another piece of the puzzle. Specifically, in the sense that even the threat of “ancestral” stimuli are learned, and their responses may differ tremendously depending on context.*

We greatly appreciate your remarks, which have helped us improve the manuscript. Additionally, thank you for summarising the manuscript’s take-home message — that is exactly what we intended to convey to the readers
