# Supplementary material for: Subjective and psychophysiological response to pictures of ancestral and modern threats: Not all evolutionary threats are alike
Source: PLoS One. 2026 Mar 18;21(3):e0343680. doi: 10.1371/journal.pone.0343680 (PMC12998808; doi:10.1371/journal.pone.0343680)
Supplement: Attachment — Submitted filename: Review_PLoS_Physiology2.docx [file pone.0343680.s014.docx]

**Reviewer #1**

*I'm satisfied with the changes made by the authors to meet my suggestions and with the motivations presented to justify the few occasions in which my suggestions have not been fully accepted.*

*I'm only concerned about a last point that I encourage to address (even if I do not consider it critical enough to prevent endorsement). In the last sentence of Conclusions (lines 732-734) is indeed written that "this is still one of the first (if not the very first) study comparing electrodermal responses across different categories of evolutionary versus modern threat stimuli": while it is one of the few studies accounting for this comparison, this is clearly not the "very first", as the authors themselves cited some articles previously comparing ancestral VS modern threats (Hugdahl & Johnsen, 1989; Flykt, Esteves & Öhman, 2007; Carlson et al., 2009; Luck et al., 2020). While the present paper integrates and enriches this research line, its "very first" nature sounds like an unnecessary overstatement: my suggestion is to entirely remove this last sentence, or at least the "(if not the very first)" specification.*

**Accepted.** We changed to last sentence which now reads “Overall, this study convincingly shows that responses may differ tremendously depending on context even for threats which are generally considered ancestral.”

**Reviewer #2**

*I would like to thank and commend the authors for their rigorous work I believe many comments have been appropriately dealt with and the manuscript reads well now. I only have a few minor points that the authors could consider, but they do not necessarily prevent the manuscript from being suitable for publication.*

*1. I find the last sentence of the conclusion (starting line 618) somewhat negative. Every study has limitations, so I would prefer a concluding sentence with more body. What are the implications of this study, how does it progress science, how does it tie in with existing literature. Maybe something similar to my suggestion in the "concluding remarks" section of my previous review if that summarizes the study succintly.*

**Accepted.** We changed to last sentence which now reads “Overall, this study convincingly shows that responses may differ tremendously depending on context even for threats which are generally considered ancestral.”

*2. The figures seem a bit disjointed; there are differences in axis width, font size, and other aesthethic elements across the figures. It would be great if the figures could be homogenized through e.g. python (matplotlib) or R to ensure the visual elements and scaling of font sizes are the same across figures. It also seems that the resolution is quite low, but that may be because of the initial submission. If not, I would recommend generating high-resolution figures.*

**Explained.** Please be assured that the figures are submitted in high resolution. We do not really understand why the editorial manager always decreases the quality to the point that it is sometimes difficult to read the plot at all… To provide an example, the smallest of the six figures is submitted with resolution of 1777x1434 pixels and all of them are exported with 300 DPI which should be more than enough regarding their content and purpose. All plots (Figures 3-6) were, in fact, plotted in R. We did some minor changes to the aesthetics in the direction you suggested. However, we believe some elements like font size are better to be changed during proofs because only at that point one can accurately judge whether a font is too small or big to the typeset size of the figure…

*2a. In figure 5, is there a reason that only the snakes are denoted with dashed lines rather than solid lines?*

**Accepted.** Yes, there is. We edited the figure caption to stress this explicitly. The relevant sentence now reads “Note that only solid lines significantly differ from zero, i.e., fear elicited by the stimuli affected the probability of electrodermal response only in heights, firearms, and airborne disease stimuli but not in snakes (dashed line).”

*3. The current implementation of the EDA processing seems to rely on peak-scoring methods. Have any other, more advanced processing pipelines such as psychophysiological modelling been considered to extract response estimates for the different stimuli?*

We extensively researched this topic about 5 years ago in collaboration with researchers from Czech Technical University. However, at that point in time, the peak-scoring methods provided much more useful insight into the collected data with regards to the hypotheses we were testing. However, new technologies emerged in the meantime, and we are revisiting this topic at the moment with a new project and new extensive data collection that is just about to start. Hence, we are open to any suggestions and would be happy to receive any specific recommendations!
